# Research progress and trends of gut microbiota and lung injury: a bibliometric and visualization analysis based on two databases (2015–2026)

**DOI:** 10.3389/fmed.2026.1902880

**Published:** 2026-10-15

**Authors:** Xinru Wu, Tiancheng Yu, Shuai Hu, Tianyi Zhang

**Affiliations:** 1 Kunshan Integrated TCM and Western Medicine Hospital, Suzhou, China; 2 School of Physical Education, Nanjing Tech University, Nanjing, China; 3 Sports and Health Collaborative Innovation Center for Fitness Promotion, Jiangsu Normal University, Xuzhou, China; 4 Institute of Physical Education, Jiangsu Normal University, Xuzhou, China

**Keywords:** bibliometric analysis, gut metabolites, gut microbiota, gut-lung axis, lung injury, probiotics, respiratory diseases, traditional Chinese medicine

## Abstract

**Background:**

Gut microbiota has emerged as an important regulator of lung injury, but the field's research structure, hotspots and emerging directions remain unclear. This study mapped the development and knowledge landscape of gut microbiota and lung injury research.

**Methods:**

Publications indexed in the Web of Science Core Collection (WoSCC) and Scopus from 2015 to May 2026 were analyzed using VOSviewer, CiteSpace and Bibliometrix.

**Results:**

Publication output increased markedly, particularly after 2019, with China and Fudan University leading national and institutional contributions, respectively. *Frontiers in Immunology* was a major publication and citation hub. Keyword patterns highlighted inflammation, the gut-lung axis, immune regulation, mouse models and sepsis.

**Conclusion:**

Major research themes included microbiota-mediated regulation of pulmonary immunity, the protective effects of short-chain fatty acids (SCFAs), and microbiota-targeted interventions involving probiotics and traditional Chinese medicine (TCM). The field remains dominated by mechanistic and preclinical studies, underscoring the need for standardized methods and clinical validation.

## 1 Introduction

Lung injury and its severe form, Acute Respiratory Distress Syndrome (ARDS), are common critical illnesses in clinical practice. They are pathologically featured by disruption of the alveolar-capillary barrier, diffuse interstitial and alveolar pulmonary edema, progressive hypoxemia and respiratory distress (1). The pathophysiological processes involve excessive inflammatory response, oxidative stress damage, alveolar epithelial cell apoptosis, disorders of the coagulation-fibrinolytic system and immune imbalance (2, 3). This disease develops rapidly with a high mortality rate. Globally, the annual incidence of ARDS is approximately 86 cases per 100,000 population, and the mortality rate of severe patients remains as high as 40%–50% (4). Sepsis, pneumonia, trauma, aspiration and severe acute pancreatitis are the most common causes of acute lung injury (ALI), among which sepsis-associated lung injury accounts for over 60% of all cases (5, 6). In addition, non-infectious lung injuries such as radiation-induced lung injury, drug-induced lung injury and ischemia-reperfusion lung injury have become increasingly prevalent clinically, posing a serious threat to patients’ lives (7, 8). Current clinical treatments for lung injury mainly include mechanical ventilation support, fluid management, anti-infection therapy and glucocorticoid administration. Lung-protective ventilation strategies are core measures to improve oxygenation and reduce mortality (9, 10). However, existing therapies are predominantly symptomatic and supportive treatments. Specific targeted drugs acting on the core pathological and physiological links of lung injury are still lacking, which fails to effectively block inflammatory cascades and progressive pulmonary damage. Moreover, long-term administration of high-dose glucocorticoids increases the risks of adverse reactions such as secondary infection and gastrointestinal bleeding (8, 11). Therefore, in-depth exploration of the pathogenesis of lung injury and identification of novel therapeutic targets and strategies have become urgent scientific issues in critical care medicine and respiratory medicine.

The gut microbiota is a complex microbial community colonizing the human gastrointestinal tract, comprising bacteria, fungi, viruses and other microorganisms. The genes encoded by gut microbiota are more than 150 times the number of human genes (12). As the largest “immune organ” and “metabolic organ” of the human body, gut microbiota plays an irreplaceable role in maintaining intestinal barrier integrity, regulating host immune responses, metabolizing nutrients and defending against pathogenic invasion (13). The gut-lung axis is a bidirectional signaling pathway connecting the gastrointestinal tract and the respiratory system. Gut microbiota modulates pulmonary immune homeostasis and inflammatory responses through this axis, with major mechanisms including entry of microbial metabolites into the blood circulation, transmucosal migration of immune cells, vagus nerve signal transduction and gut microbiota translocation (14, 15). In recent years, the regulatory role of gut microbiota and the gut-lung axis in pulmonary diseases has become a research hotspot. Accumulating evidence indicates that gut dysbiosis is closely correlated with the occurrence and progression of various pulmonary diseases (16). Previous studies have confirmed that gut microbiota and its metabolites regulate pulmonary inflammation, sustain alveolar epithelial barrier function, and modulate innate and adaptive immune responses via the gut-lung axis. These pathways are tightly associated with the onset, progression and prognosis of ALI (17, 18). Animal experiments have demonstrated that gut dysbiosis induced by broad-spectrum antibiotics markedly exacerbates ALI in mice triggered by lipopolysaccharide (LPS), cecal ligation and puncture (CLP) and oleic acid, manifested as aggravated pathological damage to lung tissues, elevated levels of inflammatory factors and increased alveolar-capillary permeability (19, 20). Conversely, supplementation with probiotics, prebiotics or fecal microbiota transplantation restores gut microbial homeostasis, alleviates inflammatory infiltration in lung tissues, and inhibits the overactivation of pro-inflammatory pathways including nuclear factor kappa B (NF-*κ*B), mitogen-activated protein kinase (MAPK) and NOD-like receptor pyrin domain-containing protein 3 (NLRP3) inflammasome, thereby improving the prognosis of animal models with lung injury (21, 22).

Clinical studies have revealed that patients with ALI/ARDS generally suffer from severe gut dysbiosis, characterized by a significant decline in microbial alpha diversity, an elevated Firmicutes/Bacteroidetes ratio, reduced abundance of beneficial bacteria such as Bifidobacterium and Lactobacillus, and overgrowth of opportunistic pathogens including Escherichia coli and Enterococcus (23, 24). The severity of gut dysbiosis is significantly correlated with patients’ oxygenation index, inflammatory factor levels, duration of mechanical ventilation and 28-day mortality (25). Furthermore, early gut microbiota intervention can reduce the incidence of ALI in critically ill patients and optimize clinical outcomes (26). Gut dysbiosis can also aggravate lung injury through the pathway of “gut-derived sepsis”. Targeting the gut-lung axis provides a novel research direction and therapeutic strategy for the prevention and treatment of lung injury (27). Existing evidence has proven that targeting gut microbiota and the gut-lung axis possesses great application potential and translational value in the management of lung injury. Nevertheless, multiple key issues remain to be addressed in this field. For instance, the specific molecular mechanisms and core metabolic targets by which gut microbiota regulates lung injury have not been fully elucidated. The characteristics of gut microbiota vary among lung injuries induced by different etiologies, so individualized microbial intervention strategies are required. The efficacy of probiotics presents obvious strain specificity and dose dependence, and unified clinical application criteria have not been established (14, 28). Meanwhile, systematic and quantitative sorting of research hotspots, evolutionary trends and knowledge maps in the field of gut microbiota and lung injury is still insufficient, which hinders researchers from quickly grasping the developmental context and future research directions.

Bibliometrics is an interdisciplinary subject that adopts mathematical, statistical and bibliographic methods to quantitatively analyze scientific literature. It can objectively and systematically reveal the development history, research hotspots, research frontiers, core authors, institutions and knowledge structure of a specific discipline (29). The application of bibliometric methods in researches on gut microbiota and lung injury can comprehensively sort out the research status and developmental context, accurately identify current research weaknesses and future directions, and provide important references and data support for subsequent basic mechanism research and clinical translation. Given the above background, this study conducted a systematic and comprehensive quantitative analysis of relevant literature on gut microbiota and lung injury retrieved from the Web of Science Core Collection (WoSCC) and Scopus databases using bibliometric approaches. It aims to address three research questions: (1) How have publication output, regional participation, institutional collaboration, and cited knowledge bases evolved from 2015 to the search cutoff date? (2) Which thematic structures and emerging topics can be stably identified across WoSCC and Scopus? (3) What database-dependent discrepancies and evidence gaps constrain current clinical translation and define priorities for future research?.

## 2 Materials and methods

### 2.1 Literature sources and search strategy

Literature retrieval was performed on May 25, 2026, in the WoSCC and Scopus databases respectively. WoSCC and Scopus were selected because they provide broad multidisciplinary coverage and structured bibliographic records, including author, affiliation, keyword, citation, and cited-reference data, that are required for bibliometric network analyses. The search fields vary depending on the database. The WoSCC subject field searches for titles, abstracts, author keywords, and additional keywords; Scopus’ TITLE-ABS-KEY field searches for titles, abstracts, and keywords. Due to the non-equivalent nature of the search scopes, the total number of publications and the frequency of keywords across the two databases cannot be directly compared. Therefore, separate analyses should be conducted for each database to verify the consistency of the core findings.

In WoSCC, the search formula was defined as: TS = (gut microb* OR intestinal microb* OR gut microflora OR intestinal microflora OR gut microorganism OR intestinal microorganism OR probiotics OR prebiotics OR synbiotics) AND TS = (acute lung injury OR lung injury*). The publication time was limited from January 1, 2015 to May 25, 2026. A total of 746 literature records were initially obtained. After restricting the document type to original research articles and reviews, the number was reduced to 705. Further screening for English literature yielded 702 records. Duplicates were removed by comparing titles and Digital Object Identifiers (DOIs), and irrelevant studies were excluded via manual screening. Finally, 702 qualified articles were retrieved from WoSCC. All literature was exported in plain text format with complete full-field information including references.

In Scopus, the search formula was defined as: TITLE-ABS-KEY (“gut microbiota” OR “gut microbiome” OR “intestinal microbiota” OR “intestinal microbiome” OR “gut flora” OR “intestinal flora”) AND TITLE-ABS-KEY (“acute Lung Injury” OR “lung injury”). Literature published between 2015 and 2026 was retrieved, generating 377 records. After filtering for original research articles and reviews, 365 records remained. A further language restriction for English resulted in 334 records. Duplicates were eliminated based on titles and DOIs, and irrelevant literature was excluded through content screening, leaving 334 qualified articles. All Scopus records were saved in CSV format with full-dimensional data including references.

### 2.2 Inclusion and exclusion criteria

This study followed established analytical frameworks from published literature (30). Origin 2018 was used to analyze annual publication trends. R software (version 4.5.1) equipped with the Bibliometrix package (version 4.01), VOSviewer (version 1.6.20) and CiteSpace (version 6.1.4) were used for subsequent bibliometric analysis. The Bibliometrix package was applied to construct and visualize scientific knowledge maps. VOSviewer was used to visualize national and institutional collaboration networks, co-citation relationships of literature and keyword co-occurrence patterns. For the collaboration network analysis based on WoSCC and Scopus, the minimum publication thresholds were set as ≥5 (countries) and ≥5 (institutions) for WoSCC, and ≥5 (countries) and ≥2 (institutions) for Scopus, to balance map integrity and readability. Research entities with scattered publications were excluded, while institutions and countries with sustained research output were retained. Sensitivity tests with thresholds of ≥3 and ≥8 were conducted, and the results showed high consistency in overall network structure, core collaboration clusters and leading countries/institutions, with only minor differences in peripheral nodes, verifying the robustness of core collaboration patterns. In co-citation analysis, the minimum citation thresholds were set as ≥20 and ≥17, with ≥25 as the control for sensitivity testing. The clusters of core references and knowledge structure remained stable, with only slight changes in low-citation literature, confirming the rationality of threshold settings. Keywords such as gut microbiota and lung injury and their synonyms were excluded to avoid interference with clustering results. CiteSpace software was used to identify the top 25 references with the strongest citation strength in this research field. The relevant parameters were set as follows: the time slice spanned 2015–2025 with a 1-year slice duration; the node type was set to cited reference; the selection criterion was top *N* = 50, and pruning was not performed. Journal Impact Factor (IF) data were derived from the 2025 Journal Citation Reports (JCR).

## 3 Results

### 3.1 Overall profile of researches on gut microbiota and lung injury

A total of 702 independent articles were obtained from WoSCC. The annual publication volume of studies on gut microbiota and lung injury showed an upward trend from 2015 to 2026. Publications entered a rapid growth phase since 2019 and peaked at 133 articles in 2025. A total of 334 independent articles were included from Scopus. The publication output remained stable with small fluctuations before 2019, followed by rapid growth after 2019. The number of publications maintained at 74 in 2025, indicating continuously increasing academic attention to this research direction. Both databases revealed a substantial rise in annual publication output between 2019 and 2025. The comparatively low figure for 2026 should not be interpreted as reduced research activity, given that only partial-year publications were captured for this year.

The highly consistent growth trends of literature in the two databases suggested that researches on gut microbiota and lung injury experienced a steady development stage in the early period and have entered a rapid development phase in recent years(Figures 1A,B).

**Figure 1:**
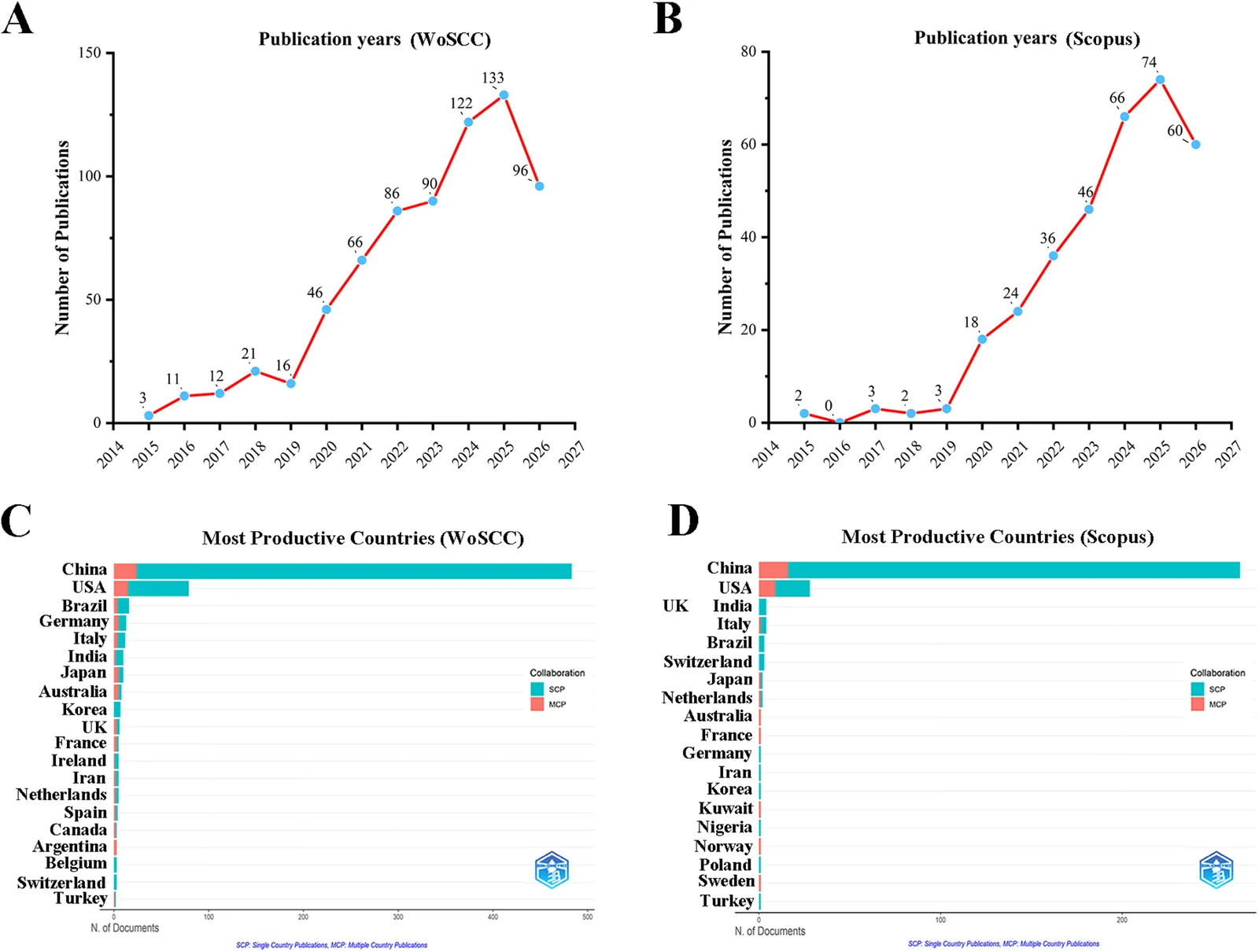
Annual publication trends on the relationship between gut microbiota and lung injury from 2015 to 2026. **(A)** Annual publication trends in WoSCC. **(B)** Annual publication trends in Scopus. **(C)** Distribution of corresponding authors’ countries in WoSCC. **(D)** Distribution of corresponding authors’ countries in Scopus.

Analysis of contributing countries showed that China ranked first globally with 483 publications in WoSCC, followed by the United States (79), Brazil (16), Germany (13) and Italy (12). Among Chinese publications, 24 involved international collaborations, accounting for 5% of the total output of China. The United States had 15 internationally collaborative articles, taking up 19% of its total publications. The results indicated that although China and the United States were the major publishing countries, international scientific collaboration in this field needs further strengthening (Table 1; Figure 1C; Figure 2A). Consistent results were observed in Scopus: China topped the list with 265 publications, followed by the United States (28), India (4), Italy (4) and Brazil (3). Sixteen publications from China were international collaborative works, accounting for 6% of its total output, which further demonstrated the insufficiency of international cooperation (Table 1; Figures 1D, 2B). Overall, China holds an absolute leading position in publication volume, while its international collaboration network needs further expansion.

**Table 1:** Most relevant countries by corresponding authors.

Country	Articles	SCP	MCP	Freq (%)	MCP_Ratio (%)
WoSCC					
China	483	459	24	68.8	5
USA	79	64	15	11.3	19
Brazil	16	12	4	2.3	25
Germany	13	8	5	1.9	38.5
Italy	12	8	4	1.7	33.3
India	10	8	2	1.4	20
Japan	10	5	5	1.4	50
Australia	8	3	5	1.1	62.5
Korea	7	7	0	1	0
United Kingdom	6	3	3	0.9	50
Scopus					
China	265	249	16	79.3	6
USA	28	19	9	8.4	32.1
India	4	4	0	1.2	0
Italy	4	3	1	1.2	25
Brazil	3	3	0	0.9	0
Switzerland	3	3	0	0.9	0
Japan	2	1	1	0.6	50
Netherlands	2	1	1	0.6	50
Australia	1	0	1	0.3	100
France	1	0	1	0.3	100

MCP, multiple country publication; SCP, single country publication.

**Figure 2:**
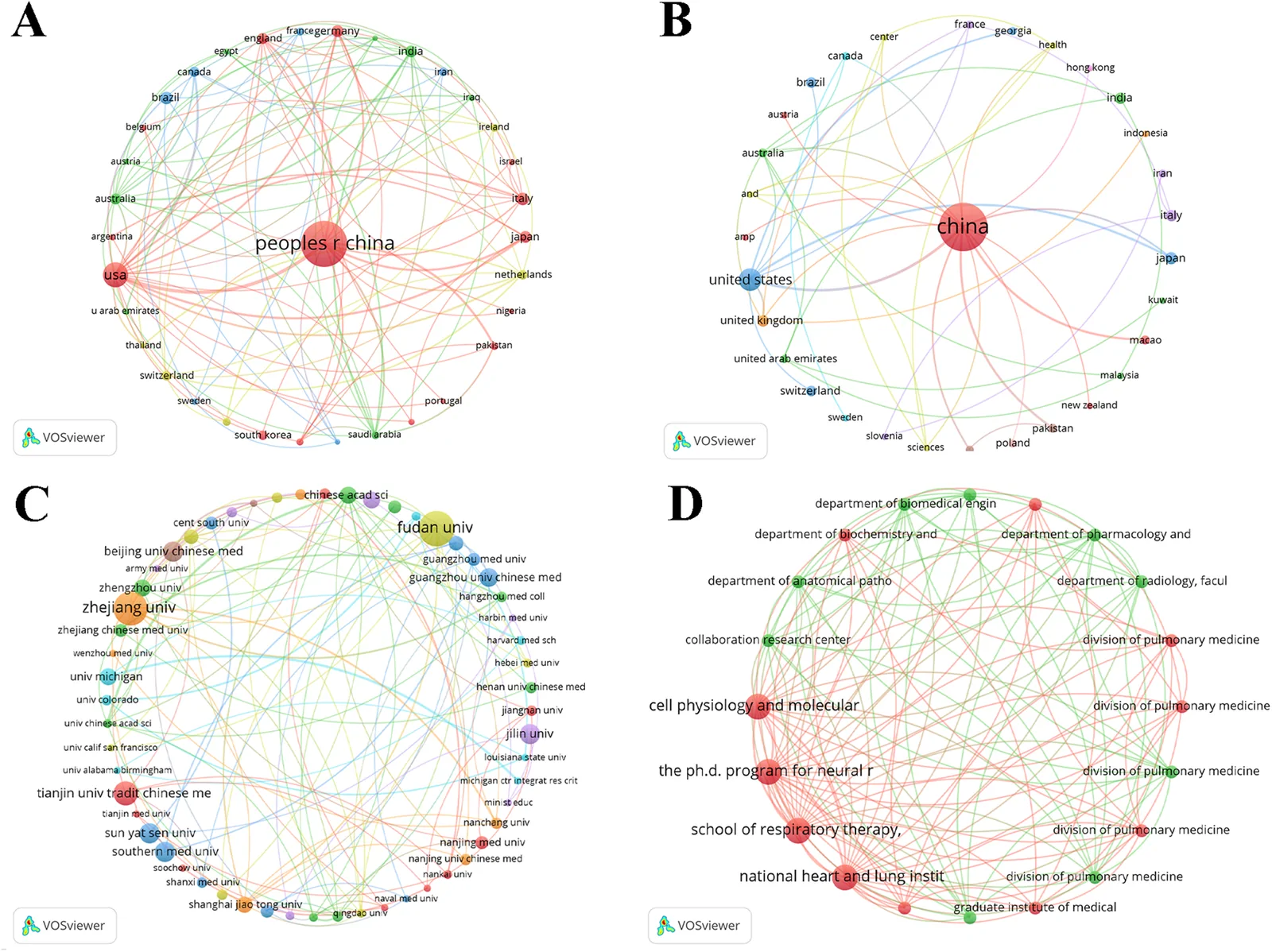
Global mapping of countries regions and institutions in gut microbiota and lung injury research from 2015 to 2026. **(A)** Geospatial collaboration network of countries based on WoSCC. **(B)** Geospatial collaboration network of countries based on Scopus. **(C)** Inter-institutional collaboration network derived from WoSCC. **(D)** Inter-institutional collaboration network derived from Scopus.

Affiliation analysis was conducted to identify productive institutions and major collaboration hubs, characterize the institutional distribution of research activity, and highlight opportunities for future cross-institutional cooperation. Institutional analysis revealed that Fudan University in China had the largest number of publications in WoSCC (25 articles) and also ranked first in Scopus (26 articles). Collaboration network analysis identified Fudan University as the core hub in both databases (Table 2; Figures 2C,D). In summary, Fudan University not only has strong research productivity but also has established extensive international collaboration networks in this field.

**Table 2:** Top 10 most relevant affiliations of gut microbiota and lung injury.

WoSCC		Scopus	
Affiliation	Articles (n)	Affiliation	Articles (n)
Fudan University	25	Fudan University	26
Zhejiang University	24	Guangzhou University of Chinese Medicine	17
Tianjin University of Traditional Chinese Medicine	17	Southern Medical University	14
Beijing University of Chinese Medicine	14	Capital Medical University	13
Jilin University	14	The First Affiliated Hospital of Dalian Medical University	12
Southern Medical University	14	Zhejiang University	12
Sun Yat-sen University	14	Naval Medical University	10
Guangzhou University of Chinese Medicine	13	The First Affiliated Hospital of Zhengzhou University	10
Chinese Academy of Sciences	12	Beijing University of Chinese Medicine	9
ChongQing Medical University	12	Henan University of Chinese Medicine	9

### 3.2 Journals and co-cited journals

The Bibliometrix and ggplot2 packages in R were used to analyze highly cited articles and journals, and VOSviewer was adopted for journal co-citation analysis. The 702 articles from WoSCC were distributed across 328 academic journals (Supplementary Material S1). As shown in Table 3 and Figure 3A, *Frontiers in Immunology* published the largest number of articles (34) with a 2025 IF of 5.9, followed by *Frontiers in Microbiology* (21 articles, IF = 4.5), *Frontiers in Pharmacology* (20 articles, IF = 4.8), *Journal of Ethnopharmacology* (20 articles, IF = 5.4) and *Frontiers in Cellular and Infection Microbiology* (18 articles, IF = 4.6). Table 4 and Figure 3B present the most highly cited journals in this field: *Frontiers in Immunology* (1,165 citations, IF = 5.9), *PLoS ONE* (797 citations, IF = 2.6), *International Journal of Molecular Sciences* (707 citations, IF = 4.9), *Nature* (667 citations, IF = 48.5) and *American Journal Of Respiratory And Critical Care Medicine* (630 citations, IF = 19.4).

**Table 3:** Top 10 journals with the most published articles.

WoSCC				Scopus		
Journal	Articles	Cites	IF (2025)	Journal	Articles	IF (2025)
Frontiers in Immunology	34	1165	5.9	Frontiers in Microbiology	18	4.5
Frontiers in Microbiology	21	521	4.5	Frontiers in Immunology	15	5.9
Frontiers in Pharmacology	20	496	4.8	Frontiers in Pharmacology	12	4.8
Journal of Ethnopharmacology	20	458	5.4	Journal of Ethnopharmacology	10	5.4
Frontiers in Cellular and Infection Microbiology	18	353	4.6	Frontiers In Cellular and Infection Microbiology	9	4.6
Biomedicine & Pharmacotherapy	14	465	7.5	International Immunopharmacology	8	4.7
Nutrients	14	573	5.0	Ecotoxicology and Environmental Safety	7	6.1
International Immunopharmacology	11	558	4.7	Biomedicine and Pharmacotherapy	5	7.5
International Journal of Biological Macromolecules	11	411	8.5	Food and Function	5	5.4
Phytomedicine	11	290	8.3	International Journal of Biological Macromolecules	5	8.5

**Figure 3:**
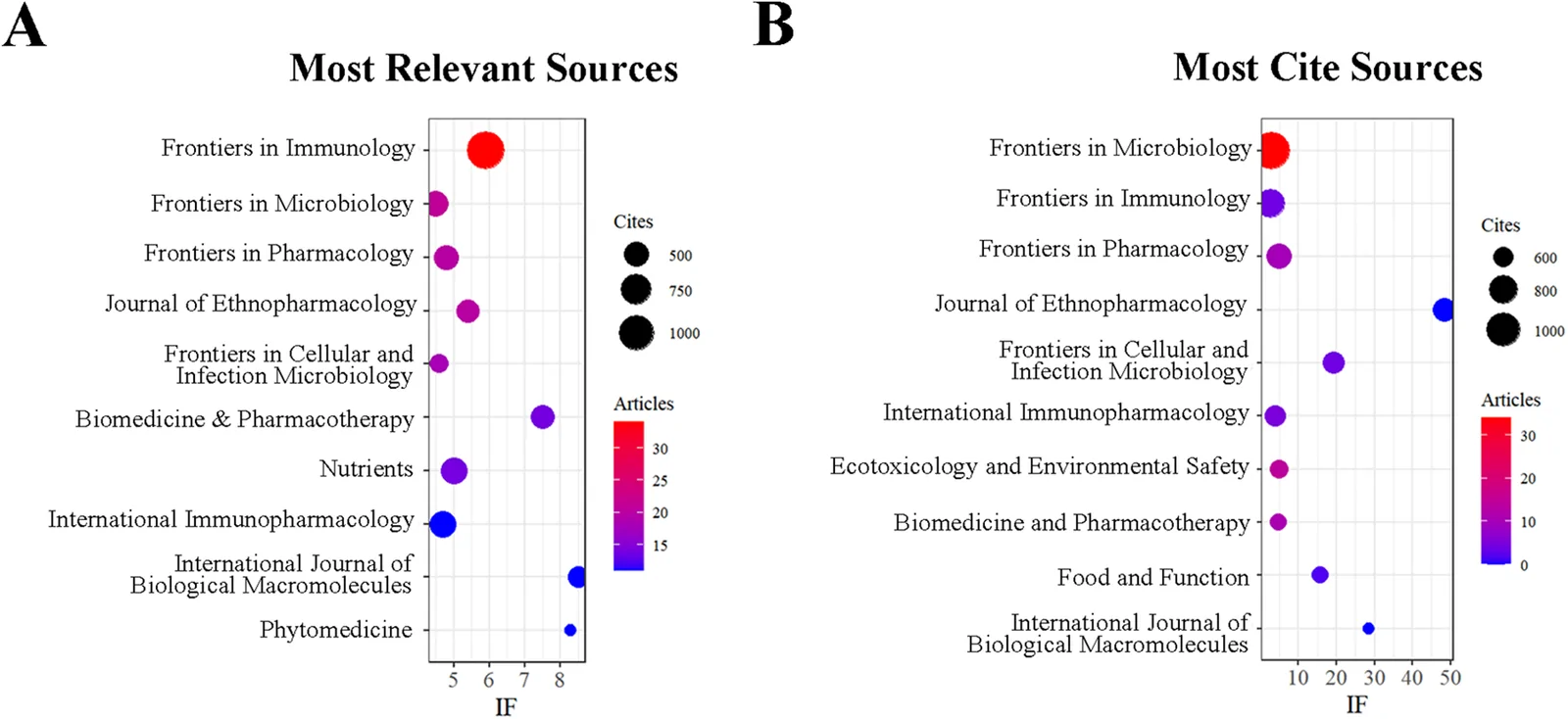
Journal with the largest number of articles published and the journal with the largest number of citations (WoSCC). **(A)** Journal with the largest number of articles published. **(B)** Journals with the largest number of citations.

**Table 4:** Top 10 journals with the most cited journals.

Rank	Journal	Cites	Articles	IF (2025)	JCR partition(2025)
1	Frontiers in Immunology	1165	34	5.9	Q1
2	PLoS ONE	797	4	2.6	Q1
3	International Journal of Molecular Sciences	707	10	4.9	Q1
4	Nature	667	0	48.5	Q1
5	American Journal Of Respiratory And Critical Care Medicine	630	4	19.4	Q1
6	Scientific Reports	618	5	3.9	Q1
7	Nutrients	573	14	5.0	Q1
8	International Immunopharmacology	558	11	4.7	Q1
9	Nature Communications	555	2	15.7	Q1
10	Gut	532	0	28.5	Q1

The 334 articles retrieved from Scopus were published in 188 journals (Supplementary Material S2). According to Table 3, *Frontiers in Microbiology* ranked first with 18 articles (IF = 4.5), followed by *Frontiers in Immunology* (15 articles, IF = 5.9), *Frontiers in Pharmacology* (12 articles, IF = 4.8) and *Journal of Ethnopharmacology* (10 articles, IF = 5.4). Journal co-citation networks based on WoSCC and Scopus (Figures 4A,B) indicated that *Frontiers in Immunology* and *PLoS ONE* acted as core hub journals. Collectively, *Frontiers in Immunology*, *Frontiers in Microbiology* and *PLoS ONE* play pivotal roles in promoting the development of researches on gut microbiota and lung injury.

**Figure 4:**
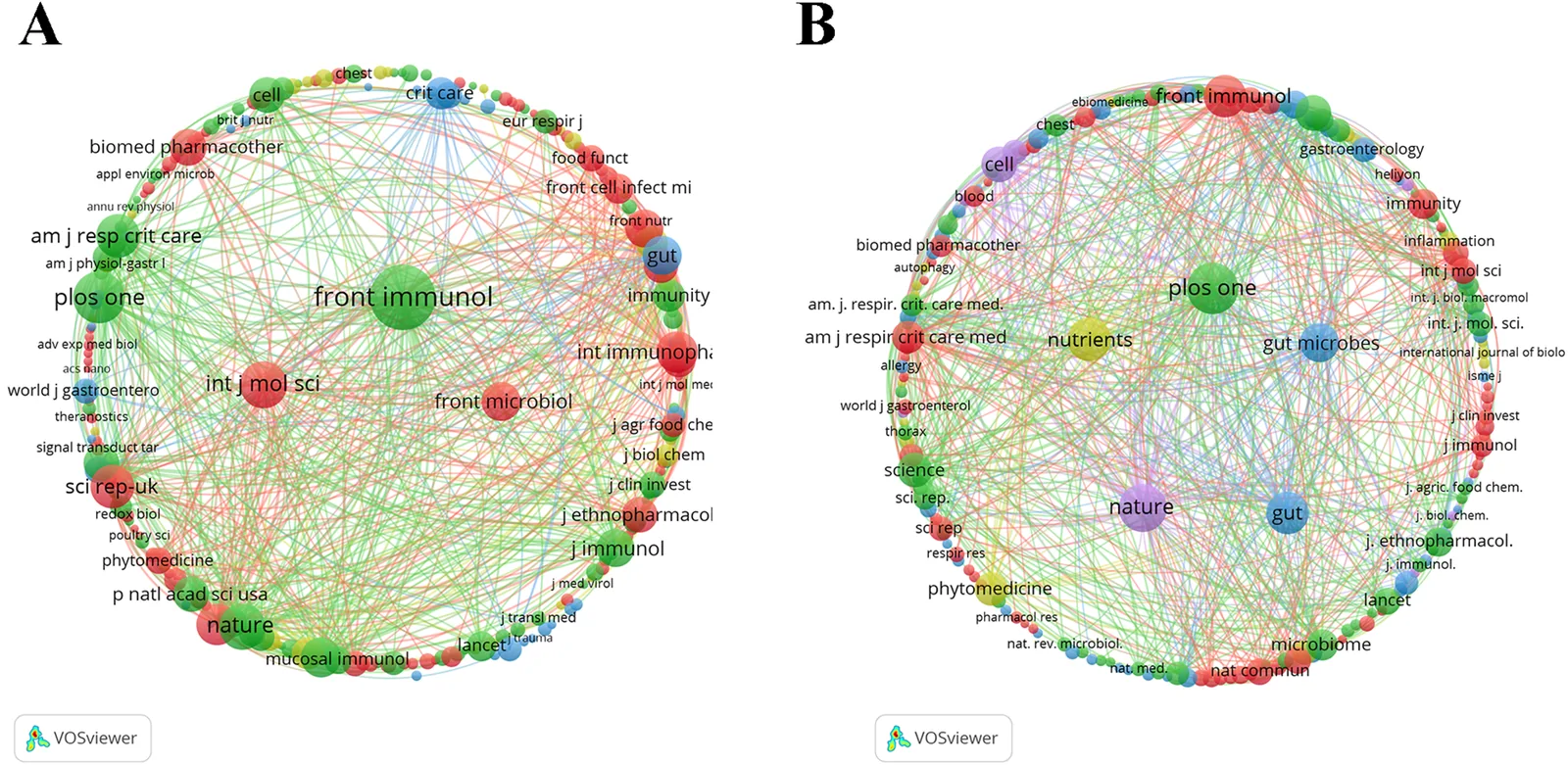
Co-cited journals related to gut microbiota and lung injury 2015 to 2026. **(A)** Journal co-citation network based on WoSCC. **(B)** Journal co-citation network based on Scopus.

### 3.3 Most cited references and citation bursts

In the WoSCC dataset, the top 25 cited references all received more than 110 citations and were published in 24 different journals (Table 5), indicating a relatively scattered citation pattern across journals. Representative high-impact articles included The microbial metabolite desaminotyrosine protects from influenza through type I interferon, Innate immune responses to trauma and Effect of gut microbiota on LPS-induced acute lung injury by regulating the TLR4/NF-kB signaling pathway.

**Table 5:** Top 25 cited references related to gut microbiota and lung injury (WoSCC).

Paper	DOI	Total Citations	TC per Year
STEED AL, 2017, SCIENCE	10.1126/science.aam5336	462	46.20
HUBER-LANG M, 2018, NAT IMMUNOL	10.1038/s41590-018-0064-8	456	50.67
TANG J, 2021, INT IMMUNOPHARMACOL	10.1016/j.intimp.2020.107272	416	69.33
HUFFNAGLE GB, 2017, MUCOSAL IMMUNOL	10.1038/mi.2016.108	401	40.10
STANLEY D, 2016, NAT MED	10.1038/nm.4194	375	34.09
CALDER PC, 2020, BMJ NUTR PREV HLTH	10.1136/bmjnph-2020-000085	320	45.71
DICKSON RP, 2020, AM J RESP CRIT CARE	10.1164/rccm.201907-1487OC	270	38.57
ISMAILOVA A, 2022, REV ENDOCR METAB DIS	10.1007/s11154-021-09679-5	253	50.60
KOMAROFF AL, 2023, FRONT MED-LAUSANNE	10.3389/fmed.2023.1187163	233	58.25
LEE SA, 2018, AM J KIDNEY DIS	10.1053/j.ajkd.2018.03.028	226	25.11
MRITYUNJAYA M, 2020, FRONT IMMUNOL	10.3389/fimmu.2020.570122	211	30.14
LI NC, 2021, J INFLAMM RES	10.2147/JIR.S341581	191	31.83
VIANA F, 2016, J PHYSIOL-LONDON	10.1113/JP270935	167	15.18
FRANZIN R, 2020, FRONT IMMUNOL	10.3389/fimmu.2020.574271	159	22.71
ZHANG Z, 2024, J HAZARD MATER	10.1016/j.jhazmat.2023.132503	142	47.33
CRIBBS SK, 2020, PHYSIOL REV	10.1152/physrev.00039.2018	141	20.14
ASHLEY SL, 2020, SCI TRANSL MED	10.1126/scitranslmed.aau9959	141	20.14
GIRON LB, 2021, FRONT IMMUNOL	10.3389/fimmu.2021.686240	141	23.50
DING ZH, 2020, PHARMACOL RES	10.1016/j.phrs.2020.104759	134	19.14
LIU Q, 2021, AM J PHYSIOL-LUNG C	10.1152/ajplung.00421.2020	132	22.00
ZHOU X, 2021, FRONT MICROBIOL	10.3389/fmicb.2021.779620	128	21.33
TORRES-FUENTES C, 2019, FASEB J	10.1096/fj.201901433R	124	15.50
VIEIRA AT, 2016, MICROBES INFECT	10.1016/j.micinf.2015.10.008	116	10.55
LIN TL, 2020, FRONT PHARMACOL	10.3389/fphar.2020.00554	115	16.43
WANG YH, 2023, EUR RESPIR J	10.1183/13993003.00840-2022	110	27.50

In Scopus, the top 25 cited articles had over 60 citations each and were distributed in 22 journals (Table 6). Milestone publications in this field covered Effect of gut microbiota on LPS-induced acute lung injury by regulating the TLR4/NF-kB signaling pathway, Lung microbiome: new insights into the pathogenesis of respiratory diseases and Gut-lung axis: The microbial contributions and clinical implications.

**Table 6:** Top 25 cited references related to gut microbiota and lung injury（scopus）.

Paper	DOI	Total Citations	TC per Year
TANG J, 2021, INT IMMUNOPHARMACOL	10.1016/j.intimp.2020.107272	450	75.00
LI R, 2024, SIGNAL TRANSDUCT TARGET THER	10.1038/s41392-023-01722-y	319	106.33
HE Y, 2017, CRIT REV MICROBIOL	10.1080/1040841X.2016.1176988	261	26.10
CHUNXI L, 2020, J IMMUNOL RES	10.1155/2020/2340670	235	33.57
ZHANG Z, 2024, J HAZARD MATER	10.1016/j.jhazmat.2023.132503	155	51.67
ASHLEY SL, 2020, SCI TRANSL MED	10.1126/SCITRANSLMED.AAU9959	148	21.14
CRIBBS SK, 2020, PHYSIOL REV	10.1152/physrev.00039.2018	147	21.00
LIU Q, 2021, AM J PHYSIOL LUNG CELL MOL PHYSIOL	10.1152/AJPLUNG.00421.2020	136	22.67
ZHOU X, 2021, FRONT MICROBIOL	10.3389/fmicb.2021.779620	129	21.50
WANG Y-H, 2023, EUR RESPIR J	10.1183/13993003.00840-2022	107	26.75
RAN Z, 2021, ENVIRON POLLUT	10.1016/j.envpol.2020.115987	101	16.83
KUVAT N, 2020, CLIN RESPIR J	10.1111/crj.13175	99	14.14
QIU F-S, 2023, BIOMED PHARMACOTHER	10.1016/j.biopha.2023.115007	94	23.50
COLE-JEFFREY CT, 2015, J CARDIOVASC PHARMACOL	10.1097/FJC.0000000000000307	86	7.17
COUTINHO-WOLINO KS, 2022, NUTR RES	10.1016/j.nutres.2022.09.001	84	16.80
WANG Z, 2022, PHARMACOL RES	10.1016/j.phrs.2022.106321	81	16.20
RUAN Y, 2023, J ETHNOPHARMACOL	10.1016/j.jep.2023.116503	80	20.00
TIAN X, 2019, FRONT MICROBIOL	10.3389/fmicb.2019.00159	72	9.00
CUI F, 2020, J MED FOOD	10.1089/jmf.2019.4544	71	10.14
ZHENG J, 2024, BIOMED PHARMACOTHER	10.1016/j.biopha.2024.116773	70	23.33
CHEN M-Y, 2019, CHIN J NAT MED	10.1016/S1875-5364 (19)30021-4	67	8.38
WANG Z, 2023, MICROBIOL SPECTR	10.1128/spectrum.03664-22	66	16.50
LI W, 2020, BIOMED PHARMACOTHER	10.1016/j.biopha.2020.110826	65	9.29
SHEN J, 2023, FOOD FUNCT	10.1039/d3fo04051h	64	16.00
PENG L-Y, 2021, FOOD FUNCT	10.1039/d1fo02407h	60	10.00

CiteSpace was used to identify articles with strong citation bursts (Figure 5). Citation-burst detection was restricted to complete calendar years from 2015 through 2025. The top three articles with the highest burst strength were Emerging pathogenic links between microbiota and the gut-lung axis (burst strength = 15.79), The gut microbiota plays a protective role in the host defence against pneumococcal pneumonia (burst strength = 11.45) and microbes, metabolites, and the gut-lung axis (burst strength = 11.3). The three latest burst articles were Intestinal Microbiota - An Unmissable Bridge to Severe Acute Pancreatitis-Associated Acute Lung Injury, Gut microbiota-derived succinate aggravates acute lung injury after intestinal ischaemia/reperfusion in mice and Gut-Lung Crosstalk in Sepsis-Induced Acute Lung Injury.

**Figure 5:**
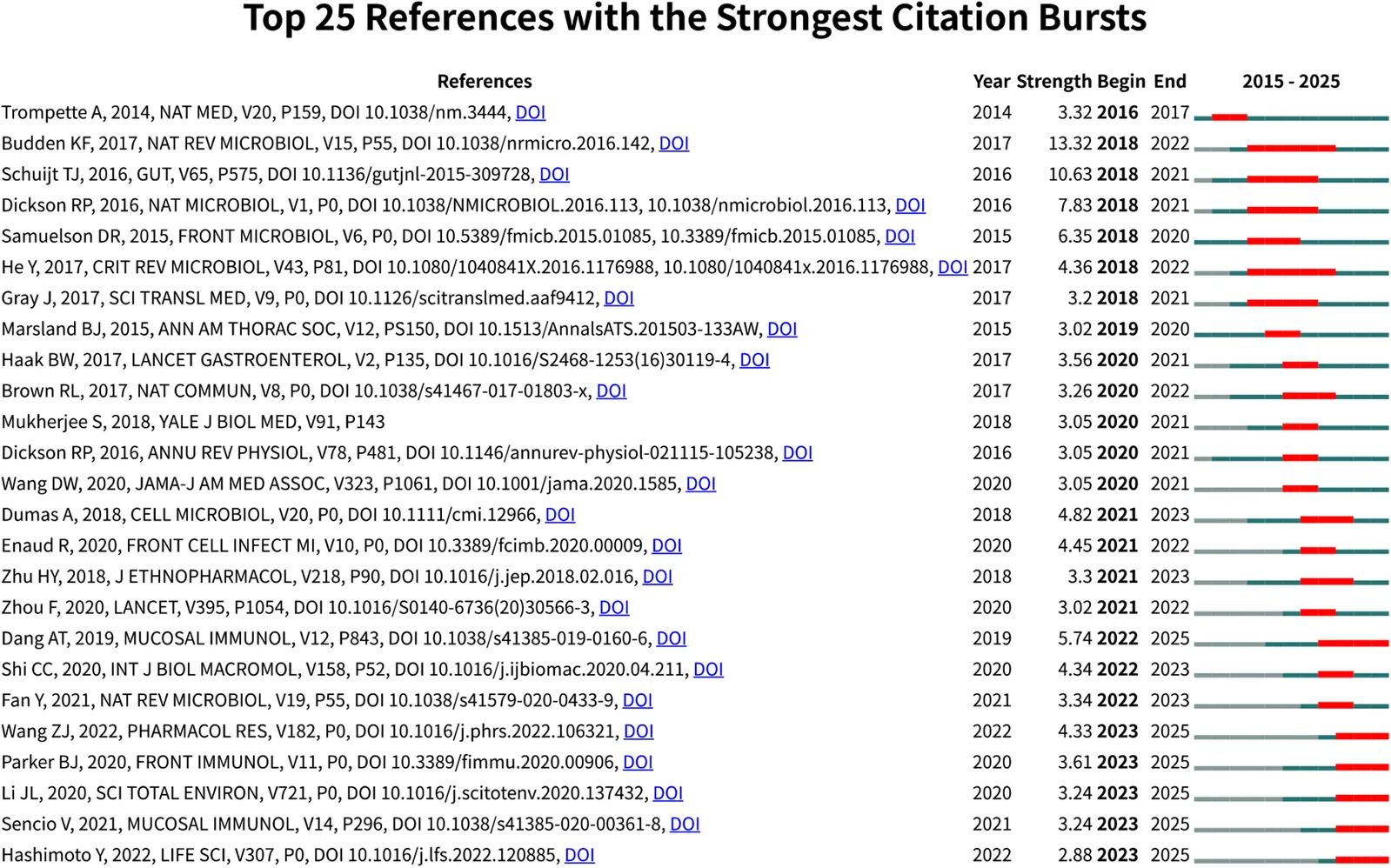
Top 25 references with the strongest citation bursts in gut microbiota and lung injury (WoSCC).

Combined analysis of cited references and citation bursts revealed that current researches mainly focus on the bidirectional regulatory mechanisms and targeted intervention strategies of the gut-lung axis centered on gut microbiota and its key metabolites. Studies systematically explore their roles, potential molecular regulatory mechanisms, values as early diagnostic biomarkers and clinical translation pathways in the occurrence, development and prognosis of ALI, ARDS, bacterial pneumonia and traumatic lung injury. The research scope includes LPS-induced ALI, sepsis-associated ALI/ARDS, pneumococcal pneumonia, lung injury caused by trauma/hemorrhagic shock, as well as lung injury complicated with intestinal barrier dysfunction, systemic inflammatory response syndrome and multiple organ dysfunction syndrome.

### 3.4 Keyword and thematic trend analysis

Keyword clustering is an effective method to identify core research topics and developmental directions. A total of 3,194 keywords were extracted from WoSCC using VOSviewer. The top 20 keywords with a frequency higher than 34 are listed in Table 7. Inflammation was the most frequent keyword (*n* = 212), followed by microbiota (*n* = 168), gut-lung axis (*n* = 97), cells (*n* = 73), mouse model (*n* = 71) and sepsis (*n* = 71). Six distinct clusters were generated via cluster analysis (Figure 6A): Red Cluster: Focuses on gut-lung axis-mediated immune regulation and microbiota-based interventions for ALI, including bacterial translocation, barrier function, innate immunity, regulatory t-cells, Th17, ARDS, critical illness, probiotics and dietary fiber. Green Cluster: Concentrates on oxidative stress and inflammatory mechanisms of gut microbiota in lung injury, covering coronavirus, NF-*κ*B, Nrf2, oxidative stress, anti-inflammation, antioxidant, chronic obstructive pulmonary disease (COPD) and pulmonary fibrosis. Blue Cluster: Explores the role of short-chain fatty acids (SCFAs)-mediated gut-lung axis regulation in inflammatory lung injury, including SCFAs, acetate, butyrate, intestinal barrier and bronchopulmonary dysplasia. Yellow Cluster: Investigates gut microbiota-mediated innate immune activation and sepsis-related lung injury, involving lipopolysaccharide, alveolar macrophages, apoptosis, extracellular vesicles and ischemia/reperfusion injury. Purple Cluster: Addresses environmental exposure-induced gut-lung microecological imbalance and airway inflammation in asthma and pulmonary fibrosis, including lung-gut axis, lung inflammation, asthma, PM2.5, epithelial cells and related responses. In total, the clustering results demonstrated that current researches have expanded from single inflammatory mechanism exploration to multiple directions, including gut-lung axis-mediated immune regulation, oxidative stress and inflammatory signaling pathways, functions of microbial metabolites such as SCFAs, mechanisms of sepsis and critical illness-related lung injury, environmental exposure-induced microecological imbalance, as well as targeted interventions including probiotics and dietary therapy.

**Table 7:** Top 20 keywords related to gut microbiota and lung injury.

WoSCC		Scopus	
Keyword	Count	Keyword	Count
inflammation	212	nonhuman	259
microbiota	168	article	214
gut-lung axis	97	animals	202
cells	73	animal experiment	197
mouse model	71	controlled study	194
sepsis	71	mouse model	194
oxidative stress	64	male	186
probiotics	60	animal tissue	181
diseases	58	metabolism	166
activation	57	mice	145
mechanisms	50	lung	144
lung	48	humans	131
infections	46	interleukin 6	123
expression	39	tumor necrosis factor	119
influenza	38	inflammation	117
nf-kappa-b	37	interleukin 1beta	104
lipopolysaccharide	37	pathology	103
metabolism	34	drug effect	101
pathogenesis	34	microbiology	100
bacteria	34	unclassified drug	94

**Figure 6:**
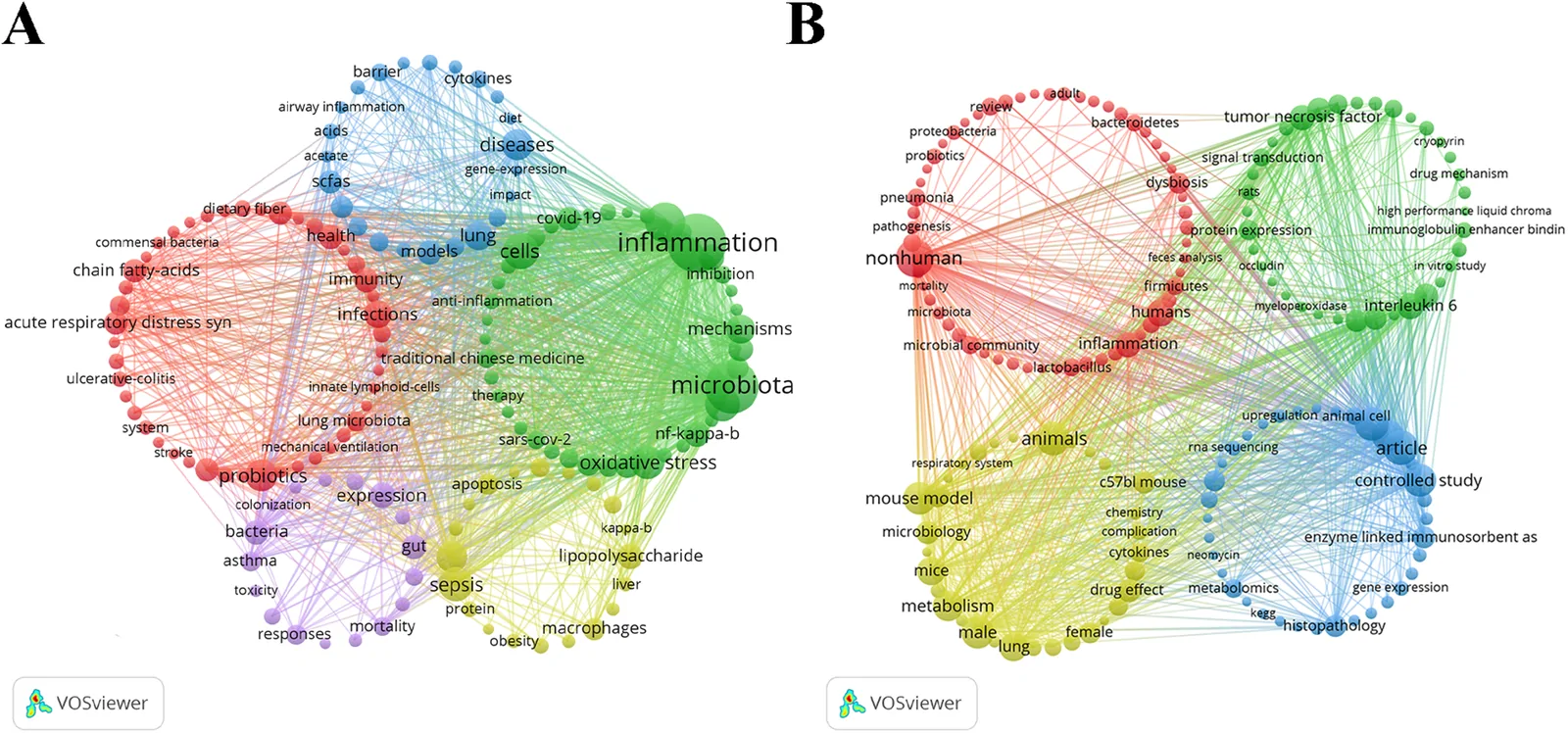
Keyword co-occurrence map of publications in gut microbiota and lung injury. **(A)** Keyword co-occurrence analysis based on WoSCC. **(B)** Keyword co-occurrence analysis based on Scopus.

A total of 4,903 keywords were extracted from Scopus. The top 20 keywords with a frequency over 94 are shown in Table 7. nonhuman ranked first (259 occurrences), followed by article (214), animals (202), animal experiment (197) and controlled study (194). Four keyword clusters were identified (Figure 6B): Red Cluster: Includes 57 keywords about immune and inflammatory regulation mediated by intestinal and pulmonary dysbiosis in chronic pulmonary diseases and sepsis-related lung injury, such as dysbiosis, microbiota, inflammation, oxidative stress, macrophage and probiotics. Green Cluster: Contains 36 keywords focusing on Toll-like receptor 4 (TLR4)/NF-*κ*B signaling pathway in LPS-induced ALI and TCM interventions, including lipopolysaccharide-induced ALI, TNF, TLR4, NF-*κ*B signaling and Chinese medicinal formula. Blue Cluster: Comprises 41 keywords related to multi-omics and high-throughput sequencing analysis of gut microbiota in infectious lung injury, such as 16S rRNA, high throughput sequencing, influenza, intestinal injury and Akkermansia. Yellow Cluster: Consists of 34 keywords highlighting the regulatory effects of SCFAs via the gut-lung axis in pulmonary diseases, including short-chain fatty acids, butyric acid, acetic acid, cytokines, mouse model and drug therapy.

To predict the future research trends in this field, we adopted the bibliometrix package of R language to map the dynamic thematic evolution. Results from WoSCC revealed that research in this area has evolved progressively from investigations into intestinal barrier function and fundamental pathological mechanisms to explorations of core inflammatory pathways and microbial structural characteristics linking gut microbiota and lung injury (Figure 7A). In recent years, it has further advanced to in-depth mechanistic analysis of the gut-lung axis and clinical translational research. Early studies (2017–2020) centered on core themes including mesenteric lymph, intestinal permeability, insulin resistance, intensive care unit, defense, resistance and mouse model. Relying primarily on animal models, these researches focused on fundamental scientific issues such as altered intestinal permeability, mesenteric lymph-associated bacterial translocation, metabolic disorders related to insulin resistance, and defensive mechanisms against lung injury in critically ill patients. They laid a solid pathophysiological foundation for subsequent studies on the correlation between gut microbiota and lung injury. During the middle phase (2021–2023), the research focus shifted to inflammation, microbiota, injury, microbiome, intestinal microbiota and lung. Scholars mainly explored gut dysbiosis, the activation of inflammatory pathways triggered by microbial metabolites, pathological progression of ALI, and immune signal transmission between the gut and lungs. These findings clarified the pivotal driving role of gut dysbiosis in the onset and progression of lung injury and established the core theoretical framework for this field. Recent studies (2024–2026) have concentrated on gut microbiota, acute lung injury, gut-lung axis, cells, acute respiratory distress syndrome and diagnosis. Research has also expanded into cutting-edge areas such as the regulation of macrophage polarization, aryl hydrocarbon receptor-mediated immunometabolic modulation, and the development of clinical diagnostic biomarkers for acute respiratory distress syndrome. This transition indicates that the field is gradually moving from basic mechanistic research toward precision medicine, which integrates gut-lung axis function restoration, specific biomarker development, and clinical diagnosis and treatment for severe lung injury.

**Figure 7:**
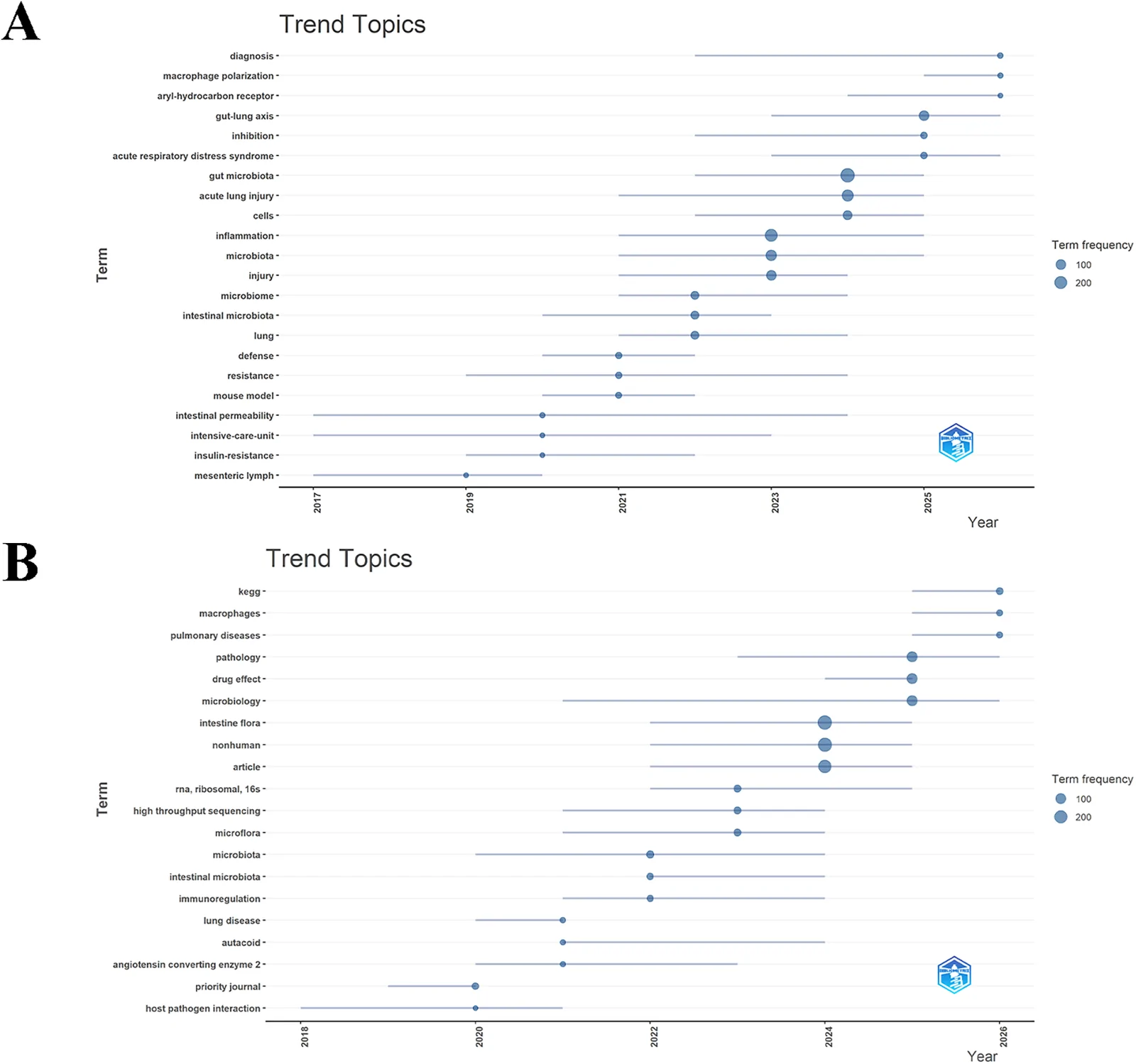
Trend topics on gut microbiota and lung injury. **(A)** Trend topics based on WoSCC. **(B)** Trend topics based on Scopus.

Analysis of data from the Scopus database (Figure 7B) demonstrated a similar evolutionary trajectory. Research topics developed from preliminary exploration of the association between gut microbiota and lung injury, to the elucidation of immune regulatory mechanisms and microbial structural features, and ultimately to in-depth research on pathological mechanisms of the gut-lung axis and the formulation of clinical translational strategies. Early studies (2018–2020) prioritized host-pathogen interaction mechanisms related to gut microbiota and lung injury, as well as the establishment of the basic literature system in this domain. High-frequency keywords included priority journal and host pathogen interaction. Using basic research models, scholars preliminarily explored the potential role of gut microbiota-mediated host-pathogen interactions in lung injury pathogenesis, thereby building the fundamental theoretical framework for follow-up investigations. The middle stage (2021–2022) focused on gut dysbiosis and the core function of microbiota-regulated immune pathways in the development of lung injury. Researchers probed the causal relationship between gut microbiota and pulmonary diseases, pathological mechanisms associated with angiotensin-converting enzyme 2 (ACE2), and the immunomodulatory effects of autacoids. Core keywords covered microbiota, intestinal microbiota, immunoregulation, lung disease, angiotensin converting enzyme 2 and autacoid. This stage clarified the core mechanism by which gut dysbiosis induces lung injury via inflammatory pathways and formed the mainstream research system of the field. From 2023 to the present, intestine flora, high throughput sequencing, 16S ribosomal RNA, microflora and pulmonary diseases have become dominant research hotspots, highlighting a trend focusing on high-throughput sequencing analysis of gut microbiota and pathological mechanism interpretation of the gut-lung axis. Emerging keywords such as pathology, drug effect, macrophages and KEGG further suggest that the field is advancing toward in-depth dissection of lung injury pathogenesis, development of targeted therapeutic drugs, and construction of precision diagnosis and treatment strategies based on multi-omics technologies.

Collectively, analyses from the two databases demonstrate that research on gut microbiota and lung injury has transitioned from early investigations into basic pathology of intestinal barrier function and host–pathogen interactions to the dissection of immune-inflammatory pathways mediated by gut dysbiosis and the establishment of the core theoretical framework of the gut–lung axis. Recent keyword trends reveal growing adoption of high-throughput sequencing, multi-omics analysis, and cell-specific mechanistic studies. These advances reflect improved methodological resolution. Nevertheless, the persistent predominance of terms related to animals, mouse models and mechanistic research indicates a substantial gap between experimental discoveries and valid patient-level applications.

### 3.5 Integrated analysis of research hotspots

Combining citation bursts, keyword clustering and thematic evolution, three core research hotspots were summarized: 1. Regulatory mechanisms of gut microbiota on the pulmonary immune microenvironment via modulating immune cell migration and barrier function; 2. Protective effects of gut microbiota-derived SCFAs against lung injury; 3. Microecological interventions for pulmonary diseases, mainly including probiotics and TCM-based strategies.

## 4 Discussion

### 4.1 Basic information

This study established a comprehensive literature dataset including 702 articles from WoSCC and 334 articles from Scopus published between 2015 and 2026. Our analyses revealed low overall publication output from 2015 to 2018 accompanied by modest inter-annual fluctuations, including the shift between 2015 and 2016. Both databases documented consistent, substantial publication growth spanning 2019-2025. Such a trend may stem from the maturing gut-lung-axis framework, broad application of high-throughput sequencing and multi-omics tools, and growing interest in host-microbiome interactions amid and following the COVID-19 pandemic. The seeming decline in 2026 publications arises mainly from the May 25, 2026 search cutoff, leading to an incomplete observation period. The included literature is predominated by preclinical, animal and mechanistic studies, while clinical-translational work stays limited. Rapid publication expansion mirrors the field's booming development and underscores an urgent need for rigorously designed clinical research to convert mechanistic findings into patient-relevant clinical evidence.

China leads globally in publication output in this field. This prominence likely stems from its sizeable biomedical research community, sustained investment in microbiome and respiratory system research, the broad adoption of sequencing and multi-omics technologies, and a strong research focus on microbiota-targeted interventions such as probiotics and traditional Chinese medicine. Importantly, publication volume is solely a metric of research activity and does not inherently equate to research quality or academic influence. Although the United States trails China in total publication count, it boasts a more extensive and structurally mature international collaboration network underpinned by its robust overall scientific research strength. The leading positions of both countries are underpinned by strong foundational research, robust policy support for scientific research, a well-established clinical translation ecosystem, and a long-standing tradition of interdisciplinary collaboration. At the institutional level, Fudan University is the most productive institution and a core hub of international collaboration. Although China has the largest publication volume, its international cooperation needs to be further strengthened, and research forces are distributed across numerous institutions, implying great potential for further development in this field. Literature was widely distributed across journals. The 702 WoSCC articles and 334 Scopus articles were published in 328 and 188 journals respectively. *Frontiers in Immunology* and *Frontiers in Microbiology* are the most productive journals, while Frontiers in Immunology and *PLoS ONE* are highly cited core journals and key nodes in co-citation networks. These journals serve as important platforms for academic communication and achievement dissemination in this field.

### 4.2 Research hotspots and trends

Integration of keyword co-occurrence, citation bursts, and thematic evolution identified a stable cross-database core rather than a collection of isolated popular terms. Immune-inflammatory regulation appeared across the WoSCC and Scopus networks, while SCFAs and microbiota-targeted interventions occupied distinct but connected thematic positions. Recent bursts and temporal keywords indicate growing interest in metabolite-mediated signaling, macrophage regulation, high-throughput sequencing, and multi-omics analysis. However, the prominence of animal, nonhuman, mouse-model, and mechanistic terms shows that this expansion remains predominantly preclinical. The principal knowledge gap is therefore not the absence of candidate mechanisms, but the limited validation of strain-specific, metabolite-specific, and dose-dependent effects in standardized human studies. Through comprehensive analysis, this study identifies three core research hotspots in the field of gut microbiota and lung injury: (1) The regulatory mechanisms of gut microbiota on the pulmonary immune microenvironment, which modulates pulmonary immunity by regulating immune cell migration and barrier function; (2) Exploring the protective effects of gut microbiota metabolites on lung injury, with short-chain fatty acids serving as key regulatory factors; (3) Microecological interventions for pulmonary diseases, with a particular focus on probiotics and traditional Chinese medicine-based therapeutic strategies.

#### 4.2.1 Regulatory mechanisms of gut microbiota on pulmonary immune microenvironment

As the largest immune organ of the human body, the intestine hosts microbiota whose interactions with immune cells not only regulate local immune homeostasis but also exert profound impacts on immune responses at distal mucosal sites via the circulatory system (31). Immune regulation mediated by the gut-lung axis modulates the pulmonary immune milieu primarily through immune cell trafficking and systemic inflammatory pathways to sustain immune homeostasis or drive inflammatory responses (32).

Upon stimulation of the intestinal mucosa by pathogenic or commensal microbial antigens, intestinal CD103⁺ dendritic cells secrete retinoic acid to upregulate the expression of homing receptors (including CCR9, CCR10 and *α*4*β*7 integrin) on lymphocytes such as IgA-producing B cells and specialized T-cell subsets, endowing these lymphocytes with the capacity to migrate toward the pulmonary mucosa (33, 34). Derived from the gut, these lymphocytes travel through the bloodstream to specifically home to bronchus-associated lymphoid tissue (BALT) and the alveolar interstitium in the lungs, where they directly participate in pulmonary immune responses by secreting IgA alongside cytokines including interferon-*γ* (IFN-*γ*) and interleukin-17 (IL-17) (35). A separate subset of gut-originated regulatory T cells (Tregs) secretes inhibitory cytokines such as interleukin-10 (IL-10) and transforming growth factor-*β* (TGF-*β*) to avert pulmonary tissue damage triggered by excessive immune activation (36, 37). Accumulating evidence also demonstrates that gut microbial metabolites prompt myeloid progenitor cells in the bone marrow to differentiate into macrophages and dendritic cells primed in a pre-activated state, which improves the recognition and clearance of pulmonary pathogens (38). Conversely, a balanced gut microbiota preserves the integrity of the intestinal epithelial barrier and prevents pathogen-associated molecular patterns (PAMPs) such as lipopolysaccharide (LPS) from entering systemic circulation (39, 40). Dysbiosis compromises gut barrier integrity to induce intestinal leakage; subsequent systemic translocation of LPS activates TLR4 and initiates systemic inflammation that exacerbates pulmonary inflammatory reactions (41, 42). Furthermore, gut dysbiosis elevates circulating levels of pro-inflammatory cytokines (IL-6, TNF-α, IL-1β among others) (43). These circulating cytokines reach the lungs and activate signaling cascades including the NF-*κ*B pathway, thereby promoting pulmonary inflammation and immune cell infiltration (44). In summary, the gut microbiota achieves refined modulation of pulmonary immunity via immune cell migration and systemic inflammatory pathways. In-depth dissection of these mechanisms facilitates clarification of the pathogeneses underlying chronic obstructive pulmonary disease, asthma, pneumonia and other pulmonary disorders, while laying novel theoretical foundations and therapeutic targets for developing microbiota-targeted interventions against lung diseases. Future investigations are warranted to characterize strain-specific regulatory effects of distinct gut bacterial strains on immune cell trafficking and activation, as well as the spatiotemporal dynamics of gut-lung immune crosstalk, so as to advance precise gut microbiota modulation for improved prevention and treatment of pulmonary illnesses.

#### 4.2.2 Protective effects of short-chain fatty acids against lung injury

SCFAs are pivotal metabolites generated from the fermentation of dietary fibers by gut microbiota, predominantly consisting of acetate, propionate and butyrate (45). The lung protective effects of SCFAs mediated by the “gut-lung axis” have become a cutting-edge direction in respiratory disease research. SCFAs show significant ameliorative effects in multiple pathological models, including ALI, acute ARDS, COPD and radiation-induced lung injury (46, 47). One of their core mechanisms is the precise regulation of pulmonary immune and inflammatory responses. SCFAs inhibit the cascade activation of pro-inflammatory signaling pathways such as NF-*κ*B and MAPK through G protein-coupled receptors GPR41, GPR43 and histone deacetylase (HDAC) inhibition pathways (48). They significantly reduce the levels of pro-inflammatory cytokines including TNF-α, IL-1β and IL-6 in bronchoalveolar lavage fluid, while upregulating the expression of anti-inflammatory cytokines such as IL-10 (49, 50). SCFAs also reshape the pulmonary immune microenvironment. They induce the polarization of alveolar macrophages toward the anti-inflammatory and reparative M2 phenotype (51), enhance the immunosuppressive function of regulatory T cells, and inhibit excessive neutrophil infiltration and abnormal activation of cytotoxic T cells (52). These effects collectively alleviate the destructive damage of inflammatory storms to lung tissue (53). In terms of barrier integrity maintenance, SCFAs promote the expression of intestinal epithelial tight junction proteins zonula occludens-1 (ZO-1) and occludin (54). They repair damaged intestinal mucosal barriers and reduce systemic inflammation caused by endotoxin and bacterial translocation. This blocks the occurrence of gut-derived lung injury at the source (54, 55). Additionally, SCFAs directly act on alveolar epithelial cells and pulmonary microvascular endothelial cells (56). They decrease vascular permeability, reduce pulmonary edema and protein exudation, and maintain the normal structure and function of the alveolar-capillary barrier (57). Furthermore, SCFAs activate the Nrf2 antioxidant signaling pathway (58). They upregulate the activities of antioxidant enzymes such as superoxide dismutase and glutathione peroxidase, scavenge excess reactive oxygen and nitrogen species, and alleviate lung cell apoptosis and necrosis induced by oxidative stress (59, 60). During chronic lung injury progression, SCFAs also inhibit the excessive activation of the TGF-*β*1/Smad signaling pathway (61). They reduce the transformation of fibroblasts into myofibroblasts and decrease the abnormal deposition of type Ⅰ and Ⅲ collagen, thereby delaying the development of pulmonary fibrosis (62). Currently, strategies including dietary fiber intervention, probiotic supplementation and SCFA aerosol administration have shown promising application prospects. However, several issues still require in-depth investigation, such as the differential effects of various SCFA types, optimal administration doses and timing, and long-term safety. These studies will provide more sufficient scientific evidence for the clinical translation of SCFAs.

#### 4.2.3 Microecological interventions for pulmonary diseases

As live microorganisms, probiotics exert pulmonary protective effects by regulating the composition of intestinal microbiota, strengthening mucosal barrier function and modulating systemic immune responses, and their therapeutic efficacy displays prominent strain specificity. Lactobacillus and Bifidobacterium represent the most extensively investigated groups of probiotics to date. Lactobacillus rhamnosus alleviates Enterococcus faecalis-triggered pulmonary inflammation by remodeling gut microbiota and restraining M1 polarization of alveolar macrophages (63). Supplemental Bifidobacterium boosts immune cell viability and restores the level of thrombospondin-1 (TSP1), facilitating the clearance of apoptotic cells and resolution of inflammation to ameliorate ALI/ARDS-associated pulmonary damage (64). Extracellular vesicles derived from Lactobacillus plantarum suppress macrophage ferroptosis and drive their polarization toward an anti-inflammatory phenotype, mitigating LPS-induced acute lung injury (65). Parabacteroides goldsteinii markedly elevates the survival rate of ALI-modeled mice, alleviates pulmonary tissue injury, and curbs the infiltration and secretion of inflammatory cells and cytokines in bronchoalveolar lavage fluid (BALF) (66). The functional mechanisms of probiotics fall into three primary categories: first, they generate metabolites including SCFAs and bile acids, which block pulmonary Toll and NF-*κ*B inflammatory signaling pathways via corresponding receptors (67); second, they modulate immune cells such as balancing T lymphocyte subsets and regulating macrophage polarization, facilitating the differentiation of Tregcells and conversion to M2 macrophages to constrain excessive inflammatory responses (51, 68); third, they reinforce epithelial barrier integrity in the intestines and lungs, limiting pathogen translocation and endotoxin leakage into the bloodstream (69, 70). During the COVID-19 pandemic, specific probiotic strains have been validated to reduce nasopharyngeal viral load, lessen pulmonary inflammatory infiltration and elevate specific antibody concentrations in patients (71).

The traditional Chinese medicine (TCM) theory of “the lung and large intestine are interior-exterior related” is highly consistent with the modern gut-lung axis concept, laying a theoretical foundation for TCM treatment of pulmonary disorders via intestinal microecology regulation (72, 73). Remarkable progress has been achieved in researches on TCM prescriptions and their bioactive constituents. It has been verified that 8*β*-Hydroxy-α-cyclocostunolide (HCC), a core bioactive ingredient from herbal medicines, exerts anti-inflammatory and antioxidant activities through modulating the Keap1-Nrf2 pathway, thereby reversing LPS-triggered pathological pulmonary alterations including inflammatory cell infiltration, pulmonary edema and alveolar collapse (74). Ruscogenin (RUS), an active component of Ophiopogon japonicus, prevents the dissociation between NMMHC IIA and TLR4, inhibits LPS-induced activation of downstream Src signaling cascades of TLR4 and degradation of adherens junction protein VE-cadherin, so as to improve pulmonary injury (75). Pudilan Anti-Inflammatory Preparation suppresses glycolysis and triggers metabolic reprogramming by inhibiting GAPDH enzymatic activity via modifying its Cys150 site, further inactivating the NF-*κ*B pathway and reducing proinflammatory cytokine release to relieve ALI (76). Compound TCM preparations embody the merits of TCM syndrome differentiation and treatment. Siji Antiviral Mixture ameliorates acute lung injury by inhibiting TNF and IL-17 signaling pathways (77).

In conclusion, as two pivotal microecological intervention modalities, probiotics and traditional Chinese medicine hold promising application prospects in the management of pulmonary injury. Probiotics feature defined chemical compositions and well-elucidated mechanisms, whereas TCM is characterized by holistic regulation and syndrome-targeted medication. Future research should focus on three orientations: firstly, large-scale, multicenter randomized controlled clinical trials ought to be conducted to clarify the applicable indications and therapeutic effects of diverse intervention regimens; secondly, in-depth exploration into the interactive mechanisms between TCM bioactive compounds and gut microbiota is required for the development of novel TCM-derived microecological preparations; thirdly, multi-omics technologies can be adopted to screen disease-specific microbial biomarkers and realize individualized precision intervention. Microecological intervention innovates therapeutic strategies for the prevention and treatment of pulmonary injury-related illnesses and is expected to serve as an essential adjunct to conventional therapeutic approaches.

### 4.3 Differences between WoSCC and scopus databases and their impacts on the identification of research hotspots

This study performs bibliometric analyses based on two databases: WoSCC and Scopus. It systematically maps the overall knowledge framework and evolutionary trends of gut microbiota and lung injury research from a complementary perspective. This study does not assume identical performance of the two databases in publication output, disciplinary coverage, and document types. Instead, it adopts a dual-database design after full consideration of their inherent structural gaps. This strategy improves the comprehensiveness and reliability of final findings. Retrieval outcomes reveal more relevant documents indexed in WoSCC than in Scopus (702 versus 334). Such numerical gaps stem from intrinsic discrepancies in journal screening criteria and subject inclusion rules. WoSCC prioritizes journals with robust academic influence and stable editorial management, favoring publications with rigorous theoretical foundations and standardized research methodologies. By contrast, Scopus covers more clinical medicine, public health and applied research. It accepts more document variants including clinical trials, observational investigations and case reports. Gut microbiota-associated lung injury represents an emerging research frontier. Current relevant work is dominated by basic animal experiments, while clinical investigations remain in infancy. Accordingly, the larger document count retrieved from WoSCC matches practical expectations.

The inclusion characteristics of the two databases are also reflected in the keyword analysis results: the six clusters from WoSCC and four clusters from Scopus do not represent a fixed number of research fields that can be directly compared. Cluster granularity depends on the size of the literature dataset, keyword sources, lexical normalization, inclusion thresholds, network density, and clustering resolution. Immunoinflammatory regulation, gut-lung axis mechanisms, microbial metabolites, and targeted microbial interventions show the strongest consistency between the two databases. Compared with the Scopus dataset, high-frequency keywords in the WoSCC dataset are mostly centered on basic research directions; keywords in the Scopus dataset tend to focus on clinical diagnosis and treatment, case observations, and trial design, including specialized clinical terms such as humans, adult population, controlled study, and clinical outcomes. The above distribution characteristics of keywords do not indicate shifts in research themes; instead, they highlight Scopus's unique strengths in including real-world clinical studies and cutting-edge exploratory research topics.

### 4.4 Research limitations

This study summarizes the status and hotspots of gut microbiome research in lung injury and identifies potential research directions, with several limitations. First, only WoSCC and Scopus were searched. Although cross-database comparison is feasible, omitting PubMed, Embase, regional databases, grey literature and non-indexed journals may miss relevant records and bias publication trends, institutional/country rankings, citation and keyword networks. Second, discrepancies between WoSCC Topic and Scopus TITLE-ABS-KEY affect retrieved records and keyword structure; numerical differences between databases cannot be fully ascribed to the underlying literature. Third, only English-language papers were included, introducing language bias. Fourth, we restricted document types to original articles and reviews, leading to incomplete literature retrieval.

## 5 Conclusion

This study summarizes the main research hotspots, emerging frontiers, knowledge context and developmental trends in the field of gut microbiota and lung injury:

- Researches on gut microbiota and lung injury have attracted global attention. China is the core research force with high academic activity, and international collaboration is expected to be further strengthened in the future.
- Frontiers in Immunology has the largest publication volume and citation count, serving as a core academic platform in this field.
- The regulatory mechanisms of gut microbiota on the pulmonary immune microenvironment have become a major research hotspot.
- The protective effects of gut microbiota-derived SCFAs against lung injury represent the mainstream research direction.
- Exploring intervention strategies of probiotics and TCM for lung injury has become a major research trend in this field.

In conclusion, this study provides reliable references for analyzing research trends and hotspots in the field of gut microbiota and lung injury. The findings help researchers quickly grasp the current status and explore future research directions. Meanwhile, the limitations and potential research directions pointed out in this paper can offer guidance for further in-depth and innovative studies.

## Data Availability

The datasets presented in this study can be found in online repositories. The names of the repository/repositories and accession number(s) can be found in the article/Supplementary Material.

## References

[1] Soumare A Kapfer T Botrel T Adda L Renaux M Blot P-L. Systemic corticosteroids, mortality, and infections in pneumonia and acute respiratory distress syndrome: a systematic review and meta-analysis. Ann Intern Med. (2026) 179(1):67–80. 10.7326/ANNALS-25-03055 41325621

[2] Harbiye A Van Den Heuvel HB Bos LDJ Boers LS. Acute respiratory distress syndrome: new pathophysiological insights. Curr Opin Crit Care. (2025) 31(5):575–81. 10.1097/MCC.0000000000001303 40621838

[3] Yang P Sjoding MW. Acute respiratory distress syndrome: definition, diagnosis, and routine management. Crit Care Clin. (2024) 40(2):309–27. 10.1016/j.ccc.2023.12.003 38432698

[4] Wick KD Ware LB Matthay MA. Acute respiratory distress syndrome. Bmj (Clinical Research Ed). (2024) 387:e76612. 10.1136/bmj-2023-076612 39467606

[5] Jia G Song E Zheng Z Qian M Liu G. Alveolar epithelial cells in bacterial sepsis-associated acute lung injury: mechanisms and therapeutic strategies. Front Immunol. (2025) 16:1605797. 10.3389/fimmu.2025.1605797 40842982 PMC12364657

[6] Wang Y Chen G Han M Ji J Wang L Dong S. Early lactate trajectories predict mortality in sepsis-associated acute lung injury: a retrospective cohort study. Respir Med. (2025) 249:108471. 10.1016/j.rmed.2025.108471 41192742

[7] Bridi GDP Fonseca EKUN Kairalla RA Amaral AF Baldi BG. Drug-induced lung disease: a narrative review. J Bras Pneumol. (2024) 50(4):e20240110. 10.36416/1806-3756/e20240110 39356911 PMC11449616

[8] Wang S Xu D Xiao L Liu B Yuan X. Radiation-induced lung injury: from mechanism to prognosis and drug therapy. Radiat Oncol. (2025) 20(1):39. 10.1186/s13014-025-02617-8 40082925 PMC11907960

[9] Chiumello D Sarma A. Lung-protective mechanical ventilation is not sex neutral. Br J Anaesth. (2026) 136(5):1436–8. 10.1016/j.bja.2026.02.007 41791986

[10] Mascia L Fanelli V Mistretta A Filippini M Zanin M Berardino M. Lung-protective mechanical ventilation in patients with severe acute brain injury: a multicenter randomized clinical trial (prolabi). Am J Respir Crit Care Med. (2024) 210(9):1123–31. 10.1164/rccm.202402-0375OC 39288368

[11] Van Kaam AH. Optimal strategies of mechanical ventilation: can we avoid or reduce lung injury?. Neonatology. (2024) 121(5):570–5. 10.1159/000539346 38870922 PMC11446299

[12] Chen Y Zhou J Wang L. Role and mechanism of gut microbiota in human disease. Front Cell Infect Microbiol. (2021) 11:625913. 10.3389/fcimb.2021.625913 33816335 PMC8010197

[13] Luo J Liang S Jin F. Gut microbiota and healthy longevity. Sci China Life Sci*.* (2024) 67(12):2590–602. 10.1007/s11427-023-2595-5 39110402

[14] Li X Shang S Wu M Song Q Chen D. Gut microbial metabolites in lung cancer development and immunotherapy: novel insights into gut-lung axis. Cancer Lett. (2024) 598:217096. 10.1016/j.canlet.2024.217096 38969161

[15] Wang L Cai Y Garssen J Henricks PAJ Folkerts G Braber S. The bidirectional gut-lung axis in chronic obstructive pulmonary disease. Am J Respir Crit Care Med. (2023) 207(9):1145–60. 10.1164/rccm.202206-1066TR 36883945 PMC10161745

[16] Ziaka M Exadaktylos A. Gut-derived immune cells and the gut-lung axis in ards. Critical Care (London, England). (2024) 28(1):220. 10.1186/s13054-024-05006-x 38965622 PMC11225303

[17] Tang J Xu L Zeng Y Gong F. Effect of gut microbiota on lps-induced acute lung injury by regulating the tlr4/nf-kb signaling pathway. Int Immunopharmacol. (2021) 91: 107272. 10.1016/j.intimp.2020.107272 33360370

[18] Xie B Wang M Zhang X Zhang Y Qi H Liu H. Gut-derived memory *γδ* t17 cells exacerbate sepsis-induced acute lung injury in mice. Nat Commun. (2024) 15(1):6737. 10.1038/s41467-024-51209-9 39112475 PMC11306781

[19] Lu Y Wu Y Huang M Chen J Zhang Z Li J. Fuzhengjiedu formula exerts protective effect against lps-induced acute lung injury via gut-lung axis. Phytomedicine. (2024) 123:155190. 10.1016/j.phymed.2023.155190 37972468

[20] Wang Y Yan Z Luo S Hu J Wu M Zhao J. Gut microbiota-derived succinate aggravates acute lung injury after intestinal ischaemia/reperfusion in mice. Eur Respir J. (2023) 61(2): 2200840. 10.1183/13993003.00840-2022 36229053

[21] Li X Ding W Lu Y Zhu H Bao W Liu Y. An anti-complement homogeneous polysaccharide from houttuynia cordata ameliorates acute pneumonia with h1n1 and mrsa coinfection through rectifying treg/th17 imbalance in the gut-lung axis and nlrp3 inflammasome activation. Acta Pharmaceutica Sinica B. (2025) 15(6):3073–91. 10.1016/j.apsb.2025.04.008 40654358 PMC12254813

[22] Wang Z Liu J Li F Ma S Zhao L Ge P. Mechanisms of qingyi decoction in severe acute pancreatitis-associated acute lung injury via gut microbiota: targeting the short-chain fatty acids-mediated ampk/nf-*κ*b/nlrp3 pathway. Microbiol Spectr. (2023) 11(4):e366422. 10.1128/spectrum.03664-22 PMC10434154 37338348

[23] Luo C Ye Y Lv A Zuo W Yang Y Jiang C. The impact of astragaloside iv on the inflammatory response and gut microbiota in cases of acute lung injury is examined through the utilization of the pi3k/akt/mtor pathway. PLoS One. (2024) 19(7):e305058. 10.1371/journal.pone.0305058 PMC11218977 38954702

[24] Shen S Gong M Wang G Dua K Xu J Xu X. COVID-19 and gut injury. Nutrients. (2022) 14(20):4409. 10.3390/nu14204409 36297092 PMC9608818

[25] Ren Z Zheng Z Feng X. Role of gut microbes in acute lung injury/acute respiratory distress syndrome. Gut Microbes. (2024) 16(1):2440125. 10.1080/19490976.2024.2440125 39658851 PMC11639474

[26] Dickson RP Singer BH Newstead MW Falkowski NR Erb-Downward JR Standiford TJ. Enrichment of the lung microbiome with gut bacteria in sepsis and the acute respiratory distress syndrome. Nat Microbiol. (2016) 1(10):16113. 10.1038/nmicrobiol.2016.113 27670109 PMC5076472

[27] Budden KF Shukla SD Bowerman KL Vaughan A Gellatly SL Wood DLA. Faecal microbial transfer and complex carbohydrates mediate protection against copd. Gut. (2024) 73(5):751–69. 10.1136/gutjnl-2023-330521 38331563

[28] De Oliveira GLV Oliveira CNS Pinzan CF De Salis LVV Cardoso CRDB. Microbiota modulation of the gut-lung axis in COVID-19. Front Immunol. (2021) 12:635471. 10.3389/fimmu.2021.635471 33717181 PMC7945592

[29] Xi X Hu S Hou W Qin Y Wu C Luo L. A bibliometric analysis of the Mediterranean diet in metabolic syndrome (2015-2025). Front Nutr. (2026) 13:1765074. 10.3389/fnut.2026.1765074 41626613 PMC12855129

[30] Liao M Cheng X Liu F Xiong X Yu Y. Bibliometric analysis of ketogenic diet for pain based on dual databases: global trends and research hotspots from the web of science core collection and scopus (2006-2025). Front Nutr. (2026) 13:1717096. 10.3389/fnut.2026.1717096 41835383 PMC12982065

[31] Sun M Lu F Yu D Wang Y Chen P Liu S. Respiratory diseases and gut microbiota: relevance, pathogenesis, and treatment. Front Microbiol. (2024) 15:1358597. 10.3389/fmicb.2024.1358597 39081882 PMC11286581

[32] Ni S Yuan X Cao Q Chen Y Peng X Lin J. Gut microbiota regulate migration of lymphocytes from gut to lung. Microb Pathog. (2023) 183:106311. 10.1016/j.micpath.2023.106311 37625662

[33] Rivera CA Randrian V Richer W Gerber-Ferder Y Delgado M-G Chikina AS. Epithelial colonization by gut dendritic cells promotes their functional diversification. Immunity. (2022) 55(1):129–44. 10.1016/j.immuni.2021.11.008 34910930 PMC8751639

[34] Schimpel C Passegger C Tam-Amersdorfer C Strobl H. Retinoic acid modulates immune differentiation in a human small intestinal *in vitro* model. Cells. (2025) 14(17):1300. 10.3390/cells14171300 40940712 PMC12428158

[35] Ocón B Xiang M Bi Y Tan S Brulois K Ayesha A. A lymphocyte chemoaffinit*y* axis for lung, non-intestinal mucosae and cns. Nature. (2024) 635(8039):736–45. 10.1038/s41586-024-08043-2 PMC11887596 39293486

[36] Jia Y He T Wu D Tong J Zhu J Li Z. The treatment of qibai pingfei capsule on chronic obstructive pulmonary disease may be mediated by th17/treg balance and gut-lung axis microbiota. J Transl Med. (2022) 20(1):281. 10.1186/s12967-022-03481-w 35729584 PMC9210581

[37] Pu Z Liu Y Chen X Ma Y Peng Y Li X. Siraitiae fructus ameliorates allergic asthma via “gut-lung axis” modulation: th17/treg ratio rebalancing, tlr4/myd88/nf-*κ*b pathway suppression, and t lymphocytes migration inhibition. Int Immunopharmacol. (2026) 171:116120. 10.1016/j.intimp.2025.116120 41485254

[38] Robles-Vera I Jarit-Cabanillas A Brandi P Martínez-López M Martínez-Cano S Rodrigo-Tapias M. Microbiota translocation following intestinal barrier disruption promotes mincle-mediated training of myeloid progenitors in the bone marrow. Immunity. (2025) 58(2):381–96. 10.1016/j.immuni.2024.12.012 39848243 PMC11832192

[39] Aja E Zeng A Gray W Connelley K Chaganti A Jacobs JP. Health effects and therapeutic potential of the gut microbe akkermansia muciniphila. Nutrients. (2025) 17(3):562. 10.3390/nu17030562 39940420 PMC11820462

[40] Liu S Cai P You W Yang M Tu Y Zhou Y. Enhancement of gut barrier integrity by a bacillus subtilis secreted metabolite through the gadd45a-wnt/*β*-catenin pathway. Imeta. (2025) 4(2):e70005. 10.1002/imt2.70005 40236773 PMC11995189

[41] Luo S Zhang Q Zhou W Zhang L Liao C Zeng D. Mettl3 promotes neutrophil extracellular trap formation via syk/erk/mek signaling during acute lung injury. J Adv Res. (2026) 23(8): 367–79. 10.1016/j.jare.2026.01.044 PMC13669593 41579954

[42] Xiang Y Zhong Y Lai X Fang Z Su G Lv Y. Myeloid-specific hdac10 deletion protects against lps-induced acute lung injury via p62 acetylation at lysine 165. Respir Res. (2024) 25(1):263. 10.1186/s12931-024-02891-2 38956592 PMC11221109

[43] Han L Sun X Kong J Li J Feng K Bai Y. Multi-omics analysis reveals a feedback loop amplifying immune responses in acute graft-versus-host disease due to imbalanced gut microbiota and bile acid metabolism. J Transl Med. (2024) 22(1):746. 10.1186/s12967-024-05577-x 39113144 PMC11308528

[44] Kumar V. Pulmonary innate immune response determines the outcome of inflammation during pneumonia and sepsis-associated acute lung injury. Front Immunol. (2020) 11:1722. 10.3389/fimmu.2020.01722 32849610 PMC7417316

[45] Mann ER Lam YK Uhlig HH. Short-chain fatty acids: linking diet, the microbiome and immunity. Nat Rev Immunol. (2024) 24(8):577–95. 10.1038/s41577-024-01014-8 38565643

[46] Chen T Mo S Shen M Du W Yu Q Chen Y. Therapeutic potential of ficus pumila l. In chronic obstructive pulmonary disease through modulation of the gut microbiota-scfa-lung signaling pathway. Food Res Int. (2026) 224:117952. 10.1016/j.foodres.2025.117952 41412647

[47] Liu J Hong W Sun Z Zhang S Xue C Dong N. The gut-lung axis: effects and mechanisms of gut microbiota on pulmonary diseases. Front Immunol. (2026) 16:1693964. 10.3389/fimmu.2025.1693964 41562083 PMC12812986

[48] Wang W Dernst A Martin B Lorenzi L Cadefau-Fabregat M Phulphagar K. Butyrate and propionate are microbial danger signals that activate the nlrp3 inflammasome in human macrophages upon tlr stimulation. Cell Rep. (2024) 43(9):114736. 10.1016/j.celrep.2024.114736 39277863

[49] Mukhopadhya I Louis P. Gut microbiota-derived short-chain fatty acids and their role in human health and disease. Nat Rev Microbiol. (2025) 23(10):635–51. 10.1038/s41579-025-01183-w 40360779

[50] Yu B Pei C Peng W Zheng Y Fu Y Wang X. Microbiota-derived butyrate alleviates asthma via inhibiting tfh13-mediated ige production. Signal Transduct Target Ther. (2025) 10(1):181. 10.1038/s41392-025-02263-2 40473603 PMC12141656

[51] Li M Pan T Yu J Wang X Gao R Wang Y. Butyrate-producing mixed probiotics alleviate mrsa-induced acute lung injury in mice by promoting m2 macrophage polarization. Probiotics Antimicrob Proteins. (2026) 18(1):541–59. 10.1007/s12602-025-10520-7 40272761

[52] Thome CD Tausche P Hohenberger K Yang Z Krammer S Trufa DI. Short-chain fatty acids induced lung tumor cell death and increased peripheral blood cd4 + T cells in nsclc and control patients *ex vivo*. Front Immunol. (2024) 15:1328263. 10.3389/fimmu.2024.1328263 38650948 PMC11033355

[53] Tjan AH Sarkissian R. The effectiveness of an internal escape channel’s role in crown cementation with various dental cements. Gen Dent. (1985) 33(3):209–11. 3896916

[54] Huang J Zhang Y Li Q Wang M Zhou H Su H. Anti-pd-l1 improves barrier function and reduces cd103(+) dc cell accumulation by regulating gut and lung microbiota and its metabolites to alleviate asthma in juvenile mice. Biomed J. (2025) 49(5):100944. 10.1016/j.bj.2025.100944 41429263 PMC13634089

[55] Liu X Shao J Liao Y Wang L Jia Y Dong P. Regulation of short-chain fatty acids in the immune system. Front Immunol. (2023) 14:1186892. 10.3389/fimmu.2023.1186892 37215145 PMC10196242

[56] Ashique S De Rubis G Sirohi E Mishra N Rihan M Garg A. Short chain fatty acids: fundamental mediators of the gut-lung axis and their involvement in pulmonary diseases. Chem Biol Interact. (2022) 368:110231. 10.1016/j.cbi.2022.110231 36288778

[57] Wu L Li Y Wang W Deng L Ge H Cui M. Gut microbiota predictive of the efficacy of consolidation immunotherapy and chemoradiotherapy toxicity in lung cancer. Med (New York, NY). (2025) 6(12):100877. 10.1016/j.medj.2025.100877 41092906

[58] Mou J Yang J Sun Y Liu J Zhao Y Lin H. An arabinogalactan from lycium barbarum mitigated dss caused intestinal injury via inhibiting mucosal damage and regulating the gut microbiota disorder. Carbohydr Polym. (2025) 352:123155. 10.1016/j.carbpol.2024.123155 39843060

[59] Fu L Cao X Liu X Xie Y Su S Zhu Y. The protective effect and mechanisms of maxing kugan decoction against oleic acid-induced acute lung injury via inhibition of inflammatory factors, oxidative damage, and modulation of intestinal microbiota and metabolites. Curr Pharm Des. (2026) 32(8):2969–85. 10.2174/0113816128383495251119071534 41574527

[60] Maruyama D Tian X Doan TNM Liao W-I Chaki T Taenaka H. Gut microbiome-derived propionate reprograms alveolar macrophages metabolically and regulates lung injury responses in mice. Gut Microbes. (2026) 18(1):2606486. 10.1080/19490976.2025.2606486 41467904 PMC12758369

[61] Li A Xiao X Feng Z Chen X Liu L Lin L. Nagilactone d ameliorates experimental pulmonary fibrosis *in vitro* and *in vivo* via modulating tgf-*β*/smad signaling pathway. Toxicol Appl Pharmacol. (2020) 389:114882. 10.1016/j.taap.2020.114882 31953203

[62] Zhu X Huo Q Jin L Du J Yu J Wang H. Parp1 stabilizes foxn3 to suppress pulmonary fibrosis through p38-related feedback regulation. Sci Adv. (2026) 12(1):eady1681. 10.1126/sciadv.ady1681 41481720 PMC12758542

[63] Li S Tong Y Li Y Sui D Li H Zhao S. Lactobacillus rhamnosus rl-h3-005 ameliorates enterococcus faecium-induced pulmonary inflammation via gut microbiota remodeling and macrophage polarization regulation. Food Res Int. (2026) 226:118239. 10.1016/j.foodres.2025.118239 41539833

[64] Kim Y Ki MS Shin MH Choi JS Park MS Kim Y. Thrombospondin-1 modulation by bifidobacterium spp. Mitigates lung damage in an acute lung injury mouse model. Microbiol Res. (2025) 297:128173. 10.1016/j.micres.2025.128173 40267843

[65] Zhao Q Lai J Jiang Y Cui E Chang H Pan R. Lactiplantibacillus plantarum -derived extracellular vesicles alleviate acute lung injury by inhibiting ferroptosis of macrophages. J Nanobiotechnology. (2025) 23(1):307. 10.1186/s12951-025-03405-y 40269965 PMC12016285

[66] Zhu W Han L He L Wei S Li J Xin L. Parabacteroides goldsteinii-derived outer membrane vesicles alleviate acute lung injury via modulation of bile acid metabolism. J Nanobiotechnology. (2026) 24(1):390. 10.1186/s12951-026-04288-3 41851729 PMC13113110

[67] Trompette A Gollwitzer ES Yadava K Sichelstiel AK Sprenger N Ngom-Bru C. Gut microbiota metabolism of dietary fiber influences allergic airway disease and hematopoiesis. Nat Med. (2014) 20(2):159–66. 10.1038/nm.3444 24390308

[68] Mitrou I Dimopoulos G Dakou K Koufargyris P Damoraki G Gkavogianni T. Interactions of probiotics with the immune cells of patients with COVID-19 pneumonia. J Innate Immun. (2025) 17(1):277–86. 10.1159/000545873 40424990 PMC12112972

[69] Li Y Yang D Zhao H Dou L Chen Q Cheng Y. The pasteurized weissella cibaria alleviates sepsis-induced acute lung injury by modulation of intestinal mucus barrier and gut microbiota. J Transl Med. (2025) 23(1):661. 10.1186/s12967-025-06674-1 40528192 PMC12172371

[70] Yao Q Liu T Wen J Yang Q Li Y Yan H. Spytag-pegylated probiotics delivering il-1ra modulate gut-lung crosstalk to mitigate septic lung injury. J Controlled Release. (2025) 386:114163. 10.1016/j.jconrel.2025.114163 40854504

[71] Wu C Xu Q Cao Z Pan D Zhu Y Wang S. The volatile and heterogeneous gut microbiota shifts of COVID-19 patients over the course of a probiotics-assisted therapy. Clin Transl Med. (2021) 11(12):e643. 10.1002/ctm2.643 34962356 PMC8713143

[72] Miao K Liu W Xu J Qian Z Zhang Q. Harnessing the power of traditional Chinese medicine monomers and compound prescriptions to boost cancer immunotherapy. Front Immunol. (2023) 14:1277243. 10.3389/fimmu.2023.1277243 38035069 PMC10684919

[73] Wang H Wang Y. What makes the gut-lung axis working? From the perspective of microbiota and traditional Chinese medicine. Can J Infect Dis Med Microbiol. (2024) 2024:8640014–15. 10.1155/2024/8640014 38274122 PMC10810697

[74] Zhang J Zhang M Zhu Q-M Xu X-R Feng Y-L Lin S. Allosteric regulation of keap1 by 8*β*-hydroxy-α-cyclocostunolide for the treatment of acute lung injury. Acta Pharmaceutica Sinica B. (2024) 14(9):4174–8. 10.1016/j.apsb.2024.06.025 39309504 PMC11413700

[75] Wu Y Yu X Wang Y Huang Y Tang J Gong S. Ruscogenin alleviates lps-triggered pulmonary endothelial barrier dysfunction through targeting nmmhc iia to modulate tlr4 signaling. Acta Pharmaceutica Sinica B. (2022) 12(3):1198–212. 10.1016/j.apsb.2021.09.017 35530141 PMC9069402

[76] Jiang M Yang Y Zhang Y Zhang P Zhang Q Yang T. Covalent targeting gapdh by pudilan formula and baicalein suppresses macrophage warburg effect to alleviate acute lung injury. Phytomedicine*.* (2026) 151:157727. 10.1016/j.phymed.2025.157727 41506101

[77] Wang Y Zhou H Wang B Liu Y Long K Zhang H. Siji kangbingdu mixture ameliorates lps-induced acute lung injury by downregulating the tnf/il-17 signaling pathways: a comprehensive study using transcriptomics, network pharmacology and experimental verification. J Inflamm Res. (2025) 18:18267–90. 10.2147/JIR.S562152 41487970 PMC12764309

